# Optimizing Exercise for Type 2 Diabetes Management: Comparative Insights from Aerobic, Resistance, Interval and Combined Training Protocols

**DOI:** 10.3390/metabo15110739

**Published:** 2025-11-12

**Authors:** Yongsheng Lan, Yujue Wang, Ruisi Wu, Ping Lv

**Affiliations:** 1 College of Physical Education, Changchun Normal University, 677 North Changji Road, Changchun 130032, China; lanys@ccsfu.edu.cn (Y.L.); qx202314005@stu.ccsfu.edu.cn (R.W.); linan@ccsfu.edu.cn (P.L.); 2 Faculty of Sports and Exercise Science, Universiti Malaya, Kuala Lumpur 50603, Malaysia

**Keywords:** type 2 diabetes, exercise modalities, insulin resistance, blood glucose regulation, GLUT4

## Abstract

**Background/Objective**: Type 2 diabetes mellitus (T2DM) is characterized by insulin resistance, β-cell dysfunction, and chronic hyperglycemia. Exercise is a cornerstone of non-pharmacological therapy, yet the optimal modalities by which different exercise prescriptions improve metabolic outcomes remain unclear. This review synthesizes evidence on the metabolic effects of aerobic, resistance, high-intensity interval (HIIT), and combined training in individuals with T2DM. **Methods**: The PubMed, Web of Science, and Scopus databases were searched up to March 30, 2025. A total of 26 articles were included. Articles were selected based on studies conducted on human participants diagnosed with type 2 diabetes mellitus, involving structured exercise interventions, and reporting at least one outcome related to insulin function or glycemic control. **Results**: This review identified five exercise programs that can improve metabolic outcomes in patients with type 2 diabetes. Evidence levels varied across the 26 studies (*n* = 20–98), so intensity ranges should be interpreted as indicative rather than prescriptive. Aerobic training was the primary intervention, and evidence from 13 studies (8–48 weeks) showed that moderate-to-vigorous intensity aerobic training (approximately 50–85% of maximum heart rate or 50–75% of VO_2_max) was generally associated with improvements in β-cell function, insulin sensitivity, and glycated hemoglobin (HbA1c). Strength training (approximately 40–50% to <3RM, 12 weeks) was linked to better glycemic parameters in some studies, though effects on insulin resistance were inconsistent. Most studies indicated that combined aerobic training (60–85% of maximum heart rate) with resistance or other complementary exercise modalities for 8–24 weeks tended to improve HbA1c, fasting glucose, and insulin sensitivity. High-intensity interval training (HIIT, ≥85% of maximum heart rate, 8 weeks) was also associated with enhanced insulin sensitivity, β-cell function, and basal insulin levels. **Conclusions**: Different exercise modalities improve metabolic health through complementary mechanisms involving enhanced glucose transport, mitochondrial function, anti-inflammatory effects, and increased muscle mass. Tailoring exercise prescriptions based on individual capacity and metabolic targets may optimize outcomes in T2DM management.

## 1. Introduction

Type 2 diabetes mellitus (T2DM) is a chronic metabolic disorder characterized by impaired insulin secretion, peripheral insulin resistance, and hyperglycemia, which together contribute to microvascular and macrovascular complications, reduced quality of life, and increased mortality [1,2]. Despite pharmacological advances, long-term glycemic control remains suboptimal in a substantial proportion of patients, highlighting the need for complementary lifestyle interventions targeting the underlying pathophysiological mechanisms of the disease.

Physical exercise has emerged as a cornerstone in the non-pharmacological management of T2DM, with compelling evidence supporting its efficacy in improving glycemic control and mitigating disease progression [3,4,5]. Regular exercise enhances glucose uptake, increases insulin sensitivity, and reduces glycated hemoglobin (HbA1c) levels [3,6,7]. These benefits are underpinned by complex metabolic adaptations in skeletal muscle, liver, and adipose tissue [8], including augmented glucose transporter type 4 (GLUT4) translocation, mitochondrial biogenesis, suppression of hepatic gluconeogenesis, and modulation of inflammatory mediators [9].

However, the optimal exercise modalities and protocols for improving specific metabolic outcomes in individuals with T2DM remain an area of active investigation. Aerobic training [4,10,11], resistance training [7,12], high-intensity interval training (HIIT) [6,13], and various combined regimens exert distinct but sometimes overlapping physiological effects [7,14], each targeting different nodes of metabolic dysfunction such as insulin resistance and β-cell dysfunction. Furthermore, differences in exercise intensity, duration, and frequency cause variation in their efficacy [15,16].

This review aims to compare different exercise prescriptions and their underlying metabolic mechanisms—namely aerobic training, resistance training, HIIT, and their combinations—in improving glycemic parameters, insulin sensitivity, pancreatic β-cell function, and HbA1c levels in individuals with T2DM, with a particular focus on AMPK–PGC-1α signaling, GLUT4 translocation, mitochondrial adaptations, and anti-inflammatory pathways. By comparing different exercise modalities, they can be personalized to tackle specific metabolic issues, and a framework for exercise-based therapeutic strategies targeting metabolic dysfunctions in diabetes care can be provided.

## 2. Materials and Methods

### Search Strategy

This systematic review was conducted in accordance with the Preferred Reporting Items for Systematic Reviews and Meta-analyses (PRISMA) Guideline [17]. The literature search was conducted across the following databases: PubMed, Web of Science, and Scopus [18]. Peer-reviewed articles published in English until 30th March 2025 were reviewed. No contact with the studies’ authors was made. The search strategy used in Scopus is displayed in Table 1 as an example, and a similar strategy was used to search the other databases. The detailed search strategies for PubMed and Web of Science are provided in the Supplementary Materials, Tables S1 and S2.

This research was registered in PROSPERO on 21 January 2025 (CRD42025641172) [19].

## 3. Result

### 3.1. Study Selection

**Figure 1:**
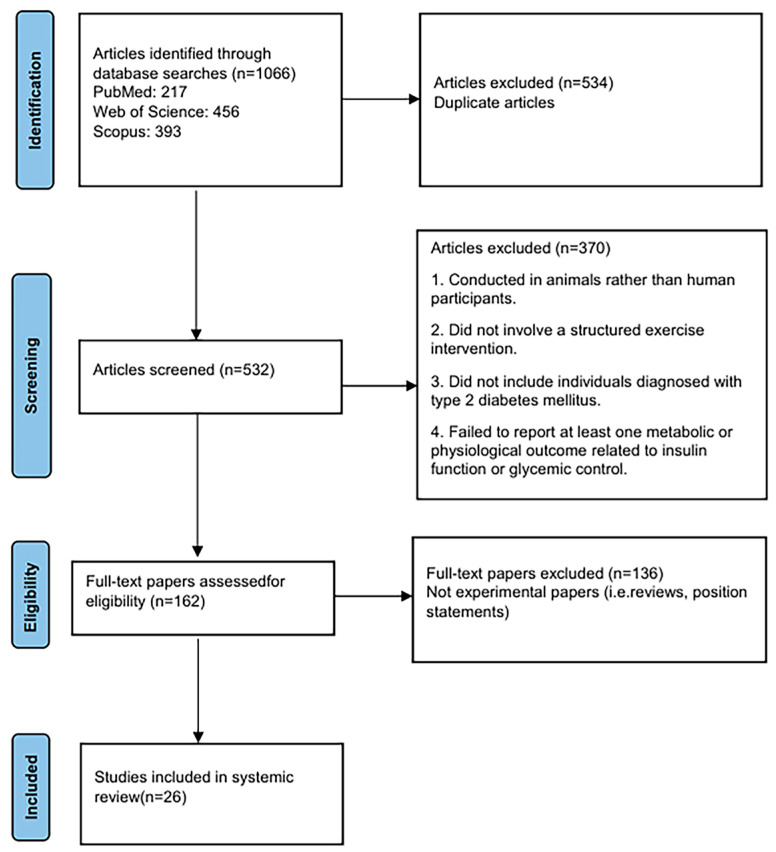
PRISMA flowchart for study selection.

Figure 1 shows a PRISMA flow diagram of the search. The articles were selected for review after thorough screening and exclusion of ineligible articles. The inclusion criteria were as follows: the article was published in English, conducted on human participants, involved a structured exercise intervention, and included individuals diagnosed with type 2 diabetes mellitus.

### 3.2. Studies Review

A total of 26 studies were included in this review, with participant ages ranging from 39 to 65 years. Baseline glycated hemoglobin (HbA1c) levels across studies ranged from 6.8% to 8.6%, indicating generally poor to moderate glycemic control among participants. The exercise interventions varied in type and intensity, including aerobic training, combined training, resistance training, and high-intensity interval training (HIIT), with intervention durations ranging from 8 to 48 weeks. Aerobic programs were typically performed at moderate-to-vigorous intensity (50–85% HRmax or 50–75% VO_2_peak), lasting 30–180 min per session, one to six times per week for 8–48 weeks; low-impact exercises such as Tai Chi were also categorized as aerobic training. Combined training protocols integrated aerobic and resistance exercises within the same session or across a weekly schedule. Resistance training, including free-weight and elastic band exercises, was generally performed three times per week for 12 weeks, at moderate-to-high intensity (40–50% to <3RM) with progressive loading (e.g., +0.5 kg per week). HIIT interventions involved repeated short bouts of exercise performed at ≥85% of maximum heart rate, interspersed with active or passive recovery periods. Table 2 presents the detailed characteristics of the included studies, including study design and participant demographics.

The papers included in the review are presented in Table 2, Table 3, Table 4 and Table 5.

### 3.3. Data Extraction and Quality Assessment

The eligibility criteria were defined according to the PICO framework [38]. Participants (P): patients diagnosed with type 2 diabetes mellitus (T2DM); Interventions (I): structured exercise training programs; Comparators (C): non-exercise control groups or alternative exercise interventions; and Outcomes (O): indicators of glucose metabolism (e.g., HbA1c, fasting glucose, or insulin sensitivity).

Two reviewers independently conducted the literature screening and data extraction. Titles and abstracts of all retrieved records were screened based on the predefined inclusion and exclusion criteria, and full texts of potentially eligible articles were reviewed in detail to confirm inclusion. Any discrepancies or disagreements between reviewers were resolved through discussion; when consensus could not be reached, a third reviewer adjudicated the decision. A small number of conflicts (n = 3) required adjudication. Inter-rater agreement was assessed qualitatively through discussion rather than statistical testing.

For each included study, data were systematically extracted on (a) participant characteristics, (b) exercise intervention parameters (frequency, intensity, duration, and type), and (c) outcome measures related to glucose metabolism. When relevant information was unclear or missing, the study authors were contacted to obtain additional details (e.g., raw data or clarification of reported outcomes). The methodological quality of the included studies was evaluated using the Cochrane risk-of-bias tool for randomized trials (RoB 2) [39].

### 3.4. Risk of Bias

Two reviewers independently assessed the risk of bias in all included randomized controlled trials using the RoB 2. Any disagreements were verified against the full text and resolved by discussion. Among the included 11 RCTs, 3 were judged as “low risk of bias” overall, while 8 were rated as having “some concerns.” The most common issues were related to deviations from the intended interventions (per-protocol effect) and potential bias in outcome measurement. No study was judged to be at “high risk of bias.”

## 4. Discussion

This review identifies 5 exercise prescriptions as effective in improving metabolic outcomes in individuals with type 2 diabetes mellitus. Aerobic training alone, when performed at a high intensity (85% of HRmax) three times per week for 8 to 12 weeks, has been shown to enhance pancreatic β-cell function, insulin-related parameters, glycemic control, and HbA1c levels [4,6]. Resistance training alone, implemented three times weekly for a minimum of 12 weeks at an intensity guided by a Borg 11–13, leads to improvements in glycemic parameters and HbA1c [3,36]. A combined HIIT (70–90% HRmax or 80–85% VO_2_peak) and resistance training (55–80% of 1 RM), performed three times per week for at least 8 weeks, has demonstrated efficacy in improving β-cell function, insulin sensitivity, and HbA1c [19]. Additionally, combined aerobic (70–89% HRmax) and resistance training (60% 1RM), performed three times per week over 12 weeks, effectively improves insulin-related indices, glycemic control, and HbA1c levels [33].

### 4.1. Effects of Aerobic Exercise on Improving Metabolic Outcomes

**Figure 2:**
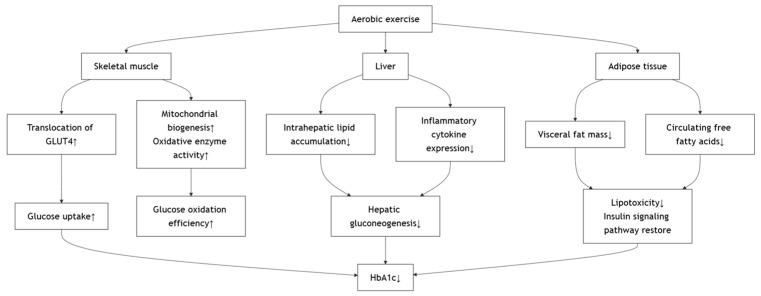
Effects of Aerobic Exercise on Improving Metabolic Outcomes.

The metabolic benefits of aerobic exercise in individuals with type 2 diabetes mellitus are mediated through multiple interrelated mechanisms. At the core of these effects is the enhancement of skeletal muscle insulin sensitivity [23,28,40], largely attributed to increased translocation of GLUT4 to the cell membrane, which facilitates greater glucose uptake and utilization [41]. This effect is known to last for up to 6 h after exercise, which leaves the muscle sensitized to insulin for up to 48 h [42]. Aerobic exercise also promotes mitochondrial biogenesis and oxidative enzyme activity in muscle tissue, thereby improving glucose oxidation efficiency and reducing reliance on anaerobic metabolism [43]. Concurrently, aerobic training suppresses hepatic gluconeogenesis by decreasing intrahepatic lipid accumulation and inflammatory cytokine expression [44], ultimately leading to reduced fasting and postprandial blood glucose levels [24,25]. These changes alleviate chronic hyperglycemia, thus lowering the availability of glucose substrates for non-enzymatic glycation of hemoglobin, reflected in decreased HbA1c levels [3,11,26,29,31,32,33,35,36]. According to the American College of Physicians (2018), maintaining HbA1c between 7% and 8% is considered clinically meaningful for most patients with type 2 diabetes, suggesting that the exercise-induced improvements observed in these studies are not only statistically significant but also clinically relevant [21]. Moreover, aerobic exercise modulates adipose tissue function by reducing visceral fat mass and circulating free fatty acids, which diminishes lipotoxicity and restores insulin signaling pathways [45]. The anti-inflammatory effects of regular aerobic activity, marked by reduced levels of C-reactive protein (CRP), interleukin-6 (IL-6), and tumor necrosis factor-α (TNF-α), further support β-cell preservation and improve insulin secretory dynamics [46]. It is worth noting that low-intensity aerobic modalities, such as Tai Chi, have also been examined in individuals with type 2 diabetes. For instance, a 16-week, twice-weekly Tai Chi program (HR during exercise: 83.3 ± 13.7) produced no significant changes in HbA1c or insulin resistance [21]. This finding suggests that exercise intensity and frequency are key determinants of metabolic adaptation and that insufficient training stimulus may limit measurable improvements. Collectively, these adaptive responses form the physiological basis by which aerobic exercise exerts its multifaceted effects on β-cell function, insulin sensitivity, glycemic control, and long-term glycemic markers such as HbA1c. The proposed mechanism involves several key tissues (Figure 2).

### 4.2. Effects of Resistance Training on Improving Metabolic Outcomes

**Figure 3:**
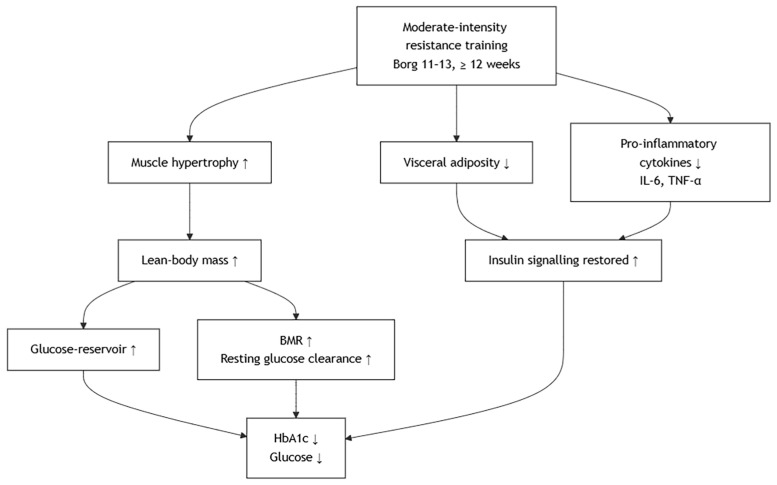
Effects of Resistance Training on Improving Metabolic Outcomes.

Compared to aerobic exercise, resistance training improves glycemic parameters and HbA1c through distinct but complementary metabolic pathways [12,32]. While aerobic training primarily enhances glucose uptake via increased oxidative capacity and mitochondrial adaptations, resistance exercise promotes muscle hypertrophy and increases lean body mass, thereby expanding the overall reservoir for glucose disposal [47]. The increased muscle mass augments basal metabolic rate and facilitates sustained glucose clearance from circulation, which is particularly relevant for reducing fasting and postprandial glycemia over time [22,48]. Resistance training also reduces visceral adiposity and attenuates chronic low-grade inflammation by lowering circulating pro-inflammatory cytokines (e.g., IL-6, TNF-α), thereby mitigating their inhibitory effects on insulin signaling [27]. Although the improvement in oxidative metabolism is less pronounced than in aerobic training, resistance exercise exerts substantial effects on glucose regulation through structural and hormonal adaptations. Nonetheless, Kwon et al. reported no improvement in insulin resistance after 12 weeks of elastic band training, indicating that low-to-moderate-intensity resistance exercise may be insufficient to induce measurable metabolic adaptations in certain diabetic populations. This finding suggests that exercise intensity and loading play a critical role in optimizing metabolic outcomes [37]. These mechanisms collectively account for the observed reductions in blood glucose and HbA1c following regular moderate-intensity resistance training, as guided by a Borg rating of 11–13 over at least 12 weeks [3]. Like aerobic training, these improvements in HbA1c are considered clinically meaningful within the target range recommended by current diabetes management guidelines. When compared to aerobic modalities, resistance training provides a muscle-centric strategy for glycemic control, and its incorporation into a combined regimen may yield additive or synergistic metabolic benefits in patients with type 2 diabetes [14,33,34]. The proposed mechanism involves several key tissues (Figure 3).

### 4.3. Effects of HIIT on Improving Metabolic Outcomes

**Figure 4:**
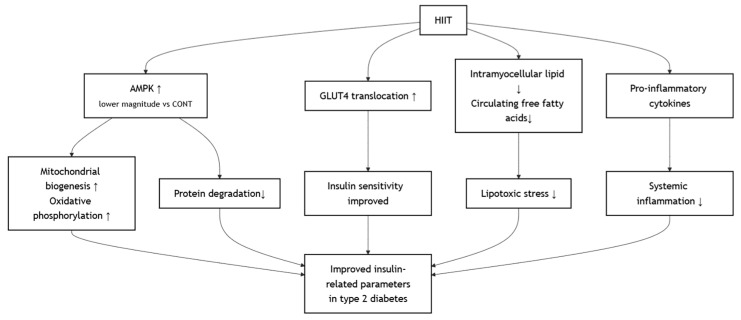
Effects of HIIT on Improving Metabolic Outcomes.

HIIT, characterized by repeated short bouts of vigorous activity interspersed with recovery periods [49], exerts unique metabolic effects that significantly improve insulin-related parameters in individuals with type 2 diabetes mellitus [6,19,33]. One of the primary mechanisms underlying these benefits is the rapid activation of the AMP-activated protein kinase (AMPK)–peroxisome proliferator-activated receptor gamma coactivator-1α (PGC-1α) signaling pathway, which enhances mitochondrial biogenesis and oxidative phosphorylation capacity in skeletal muscle [50]. This was achieved, albeit with a lower magnitude of AMPK activation compared to high-volume continuous endurance exercise, which leads to less protein degradation [51]. Furthermore, the high metabolic stress induced by HIIT upregulates GLUT4 translocation to the muscle cell membrane, improving insulin sensitivity in both fasted and fed states [52]. HIIT has also been shown to reduce intramyocellular lipid accumulation and circulating free fatty acids, thereby alleviating lipotoxicity and restoring insulin receptor signaling [53]. In addition, HIIT reduces systemic inflammation by decreasing pro-inflammatory cytokines such as Tumor Necrosis Factor-alpha (TNF-α), which is known to impair insulin action [54,55]. The proposed mechanism involves several key tissues (Figure 4).

### 4.4. Effects of Combined HIIT and Resistance Training

**Figure 5:**
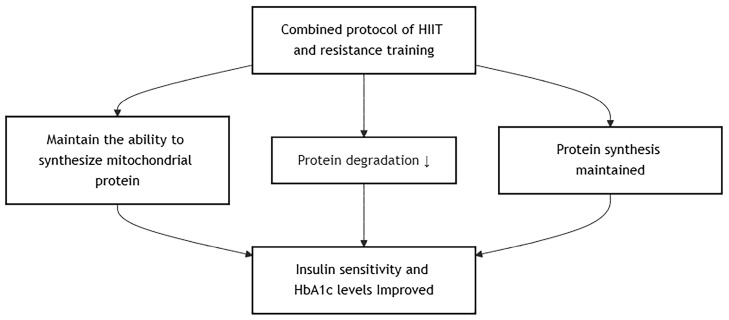
Effects of Combined HIIT and Resistance Training.

A combined protocol of HIIT and resistance training effectively improved insulin sensitivity and HbA1c levels in individuals with type 2 diabetes mellitus [19,35]. Compared to high-volume, low-intensity aerobic exercise, resistance training with HIIT still maintains the ability to synthesize mitochondrial protein while attenuating AMPK-induced protein degradation and maintaining mTOR-induced protein synthesis [56]. Considering that a diabetic individual does need to maintain their cardiovascular fitness and muscle mass for not only diabetic management but also health and fitness maintenance in general, they should choose the most beneficial method that has the least disadvantage, and it seems that the HIIT–resistance training combination can achieve that. However, only 2 studies have been conducted on diabetic patients, and more evidence should be examined before a stand can be established. In addition, HIIT may not be beginner- or sedentary-friendly, and progressive overloading of aerobic intensity should be prescribed. The proposed mechanism involves several key tissues (Figure 5).

### 4.5. Effects of Combined Aerobic and Resistance Training

**Figure 6:**
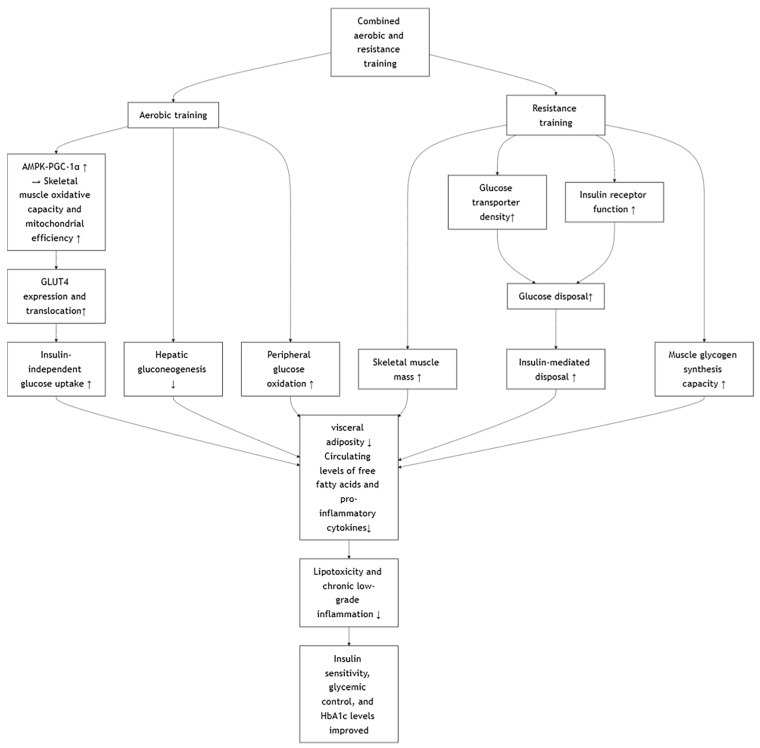
Effects of Combined Aerobic and Resistance Training.

Combined aerobic and resistance training elicits complementary physiological adaptations that contribute to significant improvements in insulin sensitivity, glycemic control, and HbA1c levels in individuals with type 2 diabetes mellitus [7,12,14,31]. Aerobic training primarily enhances skeletal muscle oxidative capacity and mitochondrial efficiency through activation of the AMPK–PGC-1α pathway [57], leading to increased GLUT4 expression and translocation, thereby facilitating insulin-independent glucose uptake [58]. Concurrently, resistance training increases skeletal muscle mass and improves glucose transporter density and insulin receptor function, expanding the capacity for insulin-mediated glucose disposal [59]. This dual-modality approach also effectively reduces visceral adiposity and circulating levels of free fatty acids and pro-inflammatory cytokines (e.g., TNF-α, IL-6), mitigating lipotoxicity and chronic low-grade inflammation [57]—two major contributors to insulin resistance and β-cell dysfunction [60]. Additionally, resistance training improves muscle glycogen synthesis capacity [61], while aerobic exercise attenuates hepatic gluconeogenesis and enhances peripheral glucose oxidation [62]. These synergistic effects result in improved fasting and postprandial glucose regulation, reduced glucose variability, and a lower glycation burden on hemoglobin over time [20,27,31,36]. However, the direct comparison between continuous aerobic exercise with HIIT in conjunction with resistance training is lacking, and thus no direct comparison can be made. It is theorized that continuous aerobic exercise, especially that with low intensity and longer duration, may diminish muscle mass [52]. Studies comparing HIIT and continuous aerobic exercise alongside resistance training should examine not only the typical diabetic outcome measures but also muscle mass and cardiovascular fitness to fully elucidate its holistic benefits to establish a stronger stand on which training method is optimal. The proposed mechanism involves several key tissues (Figure 6).

## 5. Conclusions

This review highlights the efficacy of various exercise modalities in improving metabolic health among individuals with type 2 diabetes mellitus. Aerobic, resistance, HIIT, and combined training protocols each exert distinct yet complementary effects on glycemic control, insulin sensitivity, and pancreatic β-cell function. These benefits are mediated through multiple mechanisms, including enhanced GLUT4 translocation, activation of the AMPK–PGC-1α signaling pathway, increased mitochondrial biogenesis, and reductions in inflammation and visceral adiposity.

Notably, combined exercise programs—particularly those integrating aerobic or HIIT components with resistance training—appear to produce superior outcomes by simultaneously engaging multiple metabolic pathways. Tailoring exercise prescriptions to individual capabilities, preferences, and glycemic profiles, therefore, represents a powerful, non-pharmacological approach to optimizing diabetes management.

Several key variables, including diet, medication use, baseline fitness status, and adherence to exercise programs, were insufficiently reported in many included studies. This limitation may have affected the interpretation of results and constrained the ability to determine the independent effects of exercise. In addition, the age and physical limitations of participants may influence exercise tolerance and safety, particularly for high-intensity protocols such as HIIT. Accordingly, exercise intensity should be individualized, especially for older adults or those with mobility restrictions.

The existing evidence base is further limited by variability in exercise protocols and the lack of long-term follow-up in many studies, which hinders direct comparison and assessment of the durability of observed benefits. These findings should therefore be interpreted with caution, as they are derived from heterogeneous studies with differing designs, sample sizes, and reporting standards. Additional data directly comparing continuous aerobic and HIIT protocols on glycemic and fitness outcomes are needed to clarify the optimal exercise strategies for individuals with diabetes. Furthermore, the long-term sustainability and potential risks associated with different exercise modalities—such as musculoskeletal injury or overtraining—remain insufficiently explored. Future high-quality randomized controlled trials with standardized reporting of exercise intensity and mechanistic outcomes are warranted to validate and refine these insights.

## 6. Implications for Clinical Practice and Public Health

From a clinical standpoint, the integration of individualized exercise prescriptions into standard diabetes care has the potential to substantially improve metabolic control and reduce pharmacological burden. Given the heterogeneity in patient response, exercise prescriptions should be tailored to account for baseline fitness, metabolic profiles, and comorbid conditions. Furthermore, the complementary mechanisms elicited by combined training regimens support their prioritization in clinical guidelines.

At the population level, these findings advocate for the expansion of community-based and preventive exercise programs targeting individuals with or at risk for type 2 diabetes. Examples may include community walking groups, workplace-based HIIT sessions, or structured exercise counseling integrated into primary healthcare. Embedding exercise strategies into public health frameworks may contribute to delaying disease onset, mitigating progression, and reducing the long-term burden of diabetes-related complications. As such, exercise should be regarded not only as a lifestyle recommendation but also as a clinically and biologically validated component of comprehensive diabetes management.

## Figures and Tables

**Table 1:** Search strategy for Scopus.

Filter:	(English) AND (in Title and Abstract)	Results
#1	(exercise*) AND (insulin secretion) AND (type 2 diabetes)	323
#2	(exercise*) AND (insulin secretion” OR “insulin sensitivity” OR “β-cell function” OR HOMA-IR OR HbA1c OR “glycemic control” OR “glucose control”) AND (type 2 diabetes)	169
#3	Total after merging duplicate articles from #1 and #2	393

**Table 2:** Studies reviewed on aerobic exercise.

Reference	Subjects’ Characteristics	Exercise Intervention			Main Findings
		Type	Duration, Frequency	Intensity	
Burns et al., 2007 [10]	Type 2 diabetes group: *N* = 13; 7 completed 12-week aerobic training Non-diabetic control group: *N* = 18; 14 completed 12-week aerobic training.	Aerobic	12 months; >3 h/week	33% gymnastics + 66% Nordic walking	1. HbA1c Type 2 diabetes group: decreased from 8.6 ± 1.7 to 7.1 ± 1.2% *** Non-diabetic control group: decreased from 8.7 ± 1.9 to 7.2 ± 1.4% ***
Michishita et al., 2008 [20]	Impaired glucose tolerance (IGT) group: *N* = 10; Age = 56.3 ± 8.8 years; BMI = 28.1 ± 3.4 kg/m^2^; Type 2 diabetes mellitus (DM) group: *N* = 10; Age = 58.5 ± 6.2 years; BMI = 30.2 ± 2.2 kg/m^2^; Normal glucose tolerance (NGT) group: *N* = 10; Age = 51.1 ± 8.2 years; BMI = 29.7 ± 4.2 kg/m^2^.	Aerobic	12 weeks; 1–6 times/week; 180 min/week	Cycle ergometer cycling at lactate threshold.	1. HbA1c IGT group: no change; DM group: decreased from 6.8 ± 0.4 † to 6.5 ± 0.3% NGT group: no change. 2. Fasting glucose: IGT group: no change; DM group: decreased from 129.9 ± 11.8 † to 118.6 ± 9.3 * mg/dL NGT group: no change. 3. 2 h glucose: IGT group: decreased from 161.2 ± 19.5 † to 127.8 ± 31.8 mg/dL DM group: decreased from 208.7 ± 43.1 † to 157.2 ± 47.1 * mg/dL NGT group: decreased from 120.1 ± 14.3 to 106.4 ± 16.3 * mg/dL 4. Fasting insulin: IGT group: no change DM group: no change NGT group: decreased from 17.8 ± 10.5 to 14.2 ± 8.2 * μU/mL 5. Homeostatic Model Assessment of Insulin Resistance (HOMA-IR): IGT group: no change DM group: no change NGT group: decrease from 4.24 ± 2.74 to 3.41 ± 2.30 * 6. Insulinogenic index: IGT group: increase from 0.54 ± 0.24 † to 0.99 ± 0.37 * DM group: increase from 0.44 ± 0.22 † to 0.93 ± 0.45 * NGT group: no change. 7. Insulin sensitivity index: IGT group: increase from 3.59 ± 1.14 to 4.79 ± 1.56 * DM group: increase from 2.34 ± 1.75 †, to 3.08 ± 1.29 * NGT group: increase from 3.15 ± 1.53 to 3.93 ± 1.75 *
Tsang et al., 2008 [21]	*N* = 38 (1 dropout; final *N* = 37), age = 65 ± 7.8 years; 79% women; BMI = 32.2 ± 6.3 kg/m^2^; Tai Chi group: *N* = 17; Control (sham exercise) group: *N* = 20.	Tai Chi exercise and Sham Exercise (Sham protocol included light static exercises shown to have no effect on strength, flexibility, or aerobic fitness).	16 weeks; two sessions per week; 1 h supervised sessions.	Tai Chi group: 12-movement hybrid of Sun & Yang styles; includes warm-up and cool-down; same trained instructor. Control (sham exercise) group: 1 h seated/standing calisthenics and stretching (sham exercise); minimal intensity (no resistance, isometric work, or aerobic overload).	1. Insulin resistance: Tai Chi group: no change; Control (sham exercise) group: no change; 2. HbA1c: Tai Chi group: no change; Control (sham exercise) group: no change.
Kirwan et al., 2009 [22]	14 obese patients with type 2 diabetes; Age = 63 ± 1 years; BMI = 31.9 ± 2.2 kg/m^2^.	Aerobic	On 7 consecutive days; 50–60 min of exercise	80–85% HRmax	1. Glucose disposal rate (mg/kg/min) during euglycemic clamp: 40 mU stage: increased from 1.84 ± 0.32 to 2.67 ± 0.37 * 1000 mU stage: increased from 7.57 ± 0.61 to 8.84 ± 0.56 *
Solomon et al., 2010 [4]	29 older obese men and women (*N* = 29; aged: 65 ± 1 years; BMI: 33.6 ± 1.0 kg/m^2^), divided into obese with normal glucose tolerance (*N* =16) and obese with type 2 diabetic (*N* =13).	Aerobic	Aerobic: 3 months, 5 days/week, 1 h/day Diet: 55% carbohydrate, 30% fat, and 15% protein;	80–85% HRmax	1. Glucose area under the curve (AUC): Obese with normal glucose tolerance group: no change Obese with type 2 diabetic group: decreased from 14 × 10^3^ to 12 × 10^3^ mg·dL^−1^·3h *† 2. C-Peptide AUC: Obese with normal glucose tolerance group: decreased from 1.05 × 10^3^ to 0.8 × 10^3^ mg·dL·3 h Obese with type 2 diabetic group: increased from 0.25 × 10^3^ to 0.4 × 10^3^ mg·dL·3h† 3. Oral glucose-induced insulin secretion (ΔC-pep/ΔG): Obese with normal glucose tolerance group: decreased from 32 to 18 a.u Obese with type 2 diabetic group: increased from 12 to 18 a.u † 4. β-Cell function (ΔC-Pep/ΔG ÷ IR): Obese with normal glucose tolerance group: no change Obese with type 2 diabetic group: increased from 1 to 2 a.u †
Li et al., 2012 [23]	Low-intensity group: *N* = 27; Age = 52.0 ± 1.3 years; BMI = 25.9 ± 0.6 kg/m^2^; High-intensity group: *N* = 28; Age = 50.3 ± 1.2 years; BMI = 26.1 ± 0.7 kg/m^2^;	Aerobic	12-week aerobic training, 4 sessions/week,	Low-intensity group: 50% VO_2_peak throughout, 2 × 120 kcal training in the later stage High-intensity group: 65% in weeks 3–4, 75% VO_2_peak in weeks 5–12, 2 × 120 kcal training	1. Fasting insulin: Low-intensity group: Baseline to 16–24 h: decreased from 14.3 ± 1.1 to 11.0 ± 0.9 μU/mL * 16–24 h to 15 days: no change High-intensity group: Baseline to 16–24 h: decreased from 13.7 ± 1.0 to 10.5 ± 0.9 μU/mL * 16–24 h to 15 days: no change 2. HOMA-IR: Low-intensity group: Baseline to 16–24 h: decreased from 4.8 ± 0.3 to 3.6 ± 0.2 * 16–24 h to 15 days: no change High-intensity group: Baseline to 16–24 h: decreased from 4.3 ± 0.2 to 3.3 ± 0.2 * 16–24 h to 15 days: no change
Solomon et al., 2013 [13]	105 older overweight/obese adults with impaired glucose tolerance or type 2 diabetes (aged: 61 ± 1 years, BMI: 33 ± 1 kg/m^2^)	Aerobic	12 to 16 weeks, 5 days/week, 60 min/day	75% of VO_2_max	1. β-cell disposition index (DI): Phase 1 (first 30 min of oral glucose tolerance test (OGTT)): Increased from 707 ± 64 to 1054 ± 113 a.u.; Change (Δ): +351 ± 89 Phase 2 (30 to 120 min of OGTT): Increased from 3561 ± 317 to 6491 ± 672 a.u.; Change (Δ): +2946 ± 489 2. Insulin sensitivity (mol/kg/min/pM): Increased from 0.0194 ± 0.0013 to 0.0309 ± 0.0022 **
Duvivier et al., 2017 [24]	19 patients with type 2 diabetes (13 men, 6 women), age 63 ± 9 years.	Aerobic	Each subject received 3 interventions (14 h/day of sitting, 1.1 h moderate-to-high-intensity cycling, and 4.7 h standing and light walking) sequentially under free-living conditions, each lasting 4 days.	Sitting (SIT) group: about 14 h/day of sitting; Exercise (EXE) group: about 1.1 h of sitting time replaced by moderate-to-high-intensity cycling; Sit Less (SL) group: about 4.7 h of sitting time replaced by standing (+2.5 h) and light walking (+2.2 h)	1. Glucose: EXE vs. SIT group (Decrease): 7.60 ± 0.26 vs. 7.35 ± 0.23 † SL vs. SIT group: no change SL vs. EXE group: no change 2. Insulin (pmol/L): EXE vs. SIT group: no change; SL vs. SIT group (Increase): 95 ± 14 vs. 108 ± 13 † SL vs. EXE group (Increase): 95 ± 14 vs. 102 ± 14 † 3. C-peptide (nmol/L): EXE vs. SIT group: no change; SL vs. SIT group (Increase): −0.03 ± 0.09 vs. 0.06 ± 0.08† SL vs. EXE group: no change 4. 24 h glucose AUC (min × mmol/L): EXE vs. SIT group: no change SL vs. SIT group (Increase): 10,589 ± 268 vs. 11,071 ± 334 † SL vs. EXE group: no change
Lin et al., 2017 [25]	17 patients with type 2 diabetes, Age = 48.2 ± 4.1 years	Aerobic	12 weeks; 30 min per session; 3 sessions/week	60% VO_2_max (72% HRmax)	1. Oral Glucose Tolerance Test glucose AUC: Decreased from 1925 ± 479 to 1624 to 341 (min/mmol/L) ***
Rahbar et al., 2017 [26]	30 participants (aged: 48.31 ± 5.02 years; BMI: 27.40 ± 3.65 kg/m^2^), divided into aerobic group (*N* = 15) and control group (*N* = 15).	Aerobic	8 weeks, 30 min per session, 3 times/week	50–70% HRmax	1. Fasting blood glucose: Aerobic group: Decreased from 167.54 to 130.92 (mg/dL) Control group: Decreased from 146.00 to 131.73 (mg/dL) 2. HbA1c: Aerobic group: Decreased from 7.27 to 6.62% Control group: Decreased from 7.37 to 7.03%
Shakil-ur-Rehman et al., 2017 [27]	Aerobic group: *N* = 51; Age = 53.74 ± 8.75 years; Control group: *N* = 51; Age = 55.08 ± 7.67 years.	Aerobic	25 weeks, 3 times/week	10 min/session, 30 min/week at 0° incline; each subsequent phase adds 30 min/week and 3° incline every 5 weeks.	1. Fasting Blood Glucose (mg/dL): Aerobic vs. Control group: 250.07 ± 28.23 *† vs. 281.41 ± 31.30 2. Plasma Insulin (mU/L): Aerobic vs. Control group: 8.91 ±3.83 *† vs. 14.85 ± 5.27 3. Insulin Resistance: Aerobic vs. Control group: 37.97 ± 15.58 *† vs. 70.79 ± 23.30
Nuhu et al., 2018 [28]	Aerobic exercise group: *N* = 30; Age = 39.5 years; BMI = 25.4 kg/m^2^; Control group: *N* = 30; Age = 39.0; BMI = 25.2 kg/m^2^.	Aerobic	3 times/week for 12 weeks; 20 min/session progressed to 30 min from week 5.	Bounce height 10–15 cm, 90–120 bounces/min, 40–60% HRR.	1. Insulin: Aerobic exercise group: decreased from 8.00 (6.08–9.00) to 5.60 (5.00–6.03) µiu/L * Control group: no change 2. Insulin resistance score: Aerobic exercise group: decrease from 3.49 (2.05–4.58) to 1.79 (1.56–2.21) * Control group: no change
Zhang et al., 2023 [29]	10 adults with type 2 diabetes, aged 49.00 ± 5.00 years;	Aerobic	10 weeks, 4 times/week, 45–60 min per session	70–75% VO_2_peak	1. HbA1C: Decrease from 7.7 ± 0.9% to 7.2 ± 1%. 2. Glucose tolerance test: Decreased by 18% and 15% at 90 min and 120 min

* Indicate within-group differences (* = *p* < 0.05, ** = *p* < 0.01, *** = *p* < 0.001); † indicates between-group differences (*p* < 0.05). HbA1c: Glycated hemoglobin; AUC: area under the curve; NGT: normal glucose tolerance; INT: Interval exercise training; HR: Heart Rate; OGTT: Oral Glucose Tolerance Test; VO_2_peak: Peak Oxygen Uptake; Kcal: kilocalories; HOMA-IR: Homeostatic Model Assessment of Insulin Resistance; Rd glucose: Rate of disappearance of glucose; NS: Not significant.

**Table 3:** Studies reviewed on Combined Exercise.

Reference	Subjects’ Characteristics	Exercise Intervention			Main Findings
		Type	Duration, Frequency	Intensity	
Bruce et al., 2004 [30]	Type 2 group: *N* = 7; age = 48 ± 2; BMI = 32.3 ± 2.3; Control group: *N* = 6; age = 46 ± 3; BMI = 28.8 ± 1.1.	Combined aerobic and Interval Training	8 weeks, 3 sessions per week	Weeks 1–4: Aerobic: 2 steady rides (70% VO_2_peak, up to 60 min); 1 interval session (3 min × 8 at 80% VO_2_peak, 2 min rest at ~50%) Weeks 5–8: Same structure, adjusted to new VO_2_peak (steady rides at 70%, intervals at 85%)	1. Insulin sensitivity (Whole body insulin-stimulated glucose uptake): Increased by ~30% in both groups * 2. Plasma insulin: Type 2 group: Decreased from 178.2 ± 62.1 to 127.8 ± 41.3 pmol/L *† Control group: Decreased from 78.7 ± 13.0 to 70.6 ± 13.0 pmol/L *
Tokmakidis et al., 2004 [31]	*N* = 9; Age 55.2 ± 6.7 years; BMI = 31.5 ± 3.1 kg/m^2^.	Combined aerobic and strength	4 months	Aerobic: 2×/week (Mon, Thu), 75 min/session, 60–70% HRmax (first 2 months), then 70–80% HRmax. Strength: 2×/week (Tue, Fri), 6 exercises, 3 × 12 reps at 60% 1RM, 45–60 s rest between sets, 2–3 min between exercises.	1. Fasting glucose: Baseline to 4 weeks: decreased from 144.2 ± 16.7 to 133.5 ±14.4 mg dL^−1^ * 4 weeks to 16 weeks: increased from 133.5 ± 14.4 to 137.1 ± 16.3 mg dL^−1^ * 2. HbA1c: Baseline to 4 weeks: decreased from 7.7 ± 1.7 to 7.1 ± 1.3% 4 weeks to 16 weeks: decreased from 7.1 ±1.3 to 6.9 ± 1.0% 3. Glucose AUC: Baseline to 4 weeks: decrease from 32.0 ± 4.9 to 29.4 ± 4.2 g dL^−1^ × min * 4 weeks to 16 weeks: decrease from 29.4 ± 4.2 to 28.0 ± 4.3 g dL^−1^ × min * 4. Fasting insulin: Baseline to 4 weeks: no change; 4 weeks to 16 weeks: decrease from 11.0 ± 4.0 to 9.0 ± 3.4 μU mL^−1^ * 5. Insulin AUC: Baseline to 4 weeks: decrease from 7.7 ± 2.7 to 6.1 ± 2.8 μU mL^−1^ × min × 10^3^ * 4 weeks to 16 weeks: decrease from 6.1 ± 2.8 to 4.8 ± 1.8 μU mL^−1^ × min × 10^3^ *
Glans et al., 2009 [32]	Arabian group: *N* = 17, age = 50 ± 6.6 years; BMI: 32.3 ± 3.3 kg/m^2^ Swedish group: *N* = 12, age = 49.5 ± 7.7 years; BMI: 32.8 ± 3.8 kg/m^2^	Combined aerobic and strength	6-month; 3 times/week; 45 min	Phase 1 (Week 1–12): Combined Resistance Training: Warm-up: 5 min 25 min Circuit training: (Ergometer cycling; Dumbbell bilateral alternately biceps/triceps curls; Dumbbell shoulder press; Seated bilateral row with rubber bands; Standing alternately leg extension with rubber bands; Step up) Core strengthening (abdominal and back exercises on the floor): 10 min Stretching: 5 min Phase 2 (Week 13–24): Aerobic Exercise Program: Warm-up: 5 min; Aerobic component (dance and step up): 25 min; Core strengthening (abdominal and back exercises on the floor): 10 min; Stretching: 5 min	1. HbA1C: Arabian group: baseline to 6 months: no change; Swedish group: baseline to 6 month: decreased from 6.9 ± 1.3 to 6.3 ± 1% *†
Meex et al., 2010 [12]	Control group: *N* = 20; Age = 59.0 ± 0.8 years; BMI = 29.7 ± 0.8 kg/m^2^; Type 2 diabetes group: *N* = 18; Age = 59.4 ± 1.1 years; BMI = 30.0 ± 0.8 kg/m^2^.	Combined aerobic and strength	12 weeks; Aerobic: 30 min/session, 2 sessions/week; Resistance: 1 session/week	Aerobic training: Cycling ergometer, 55% Wmax; Strength training: One set of 8 reps at 55% MVC and two sets of 8 reps at 75% MVC, targeting large muscle groups (chest press, leg extension, lat pull-down, leg press, triceps curls, biceps curls, abdominal crunches, horizontal row)	1. Plasma insulin: Basal: Control group: decreased from 18.1 ± 2.4 to 16.1 ± 2.1 mU/L * Type 2 diabetes group: decreased from 16.4 ± 1.2 to 14.6 ± 0.8 mU/L * Clamp: Control group: no change; Type 2 diabetes group: no change. 2. Glucose disposal rate: Basal: Control group: no change; Type 2 diabetes group: decrease from 11.6 ± 0.7 to 9.9 ± 0.6 μmol·kg^−1^·min^−1^ *† Clamp: Control group: no change; Type 2 diabetes group: increased from 18.4 ± 1.4 to 21.0 ± 1.4 μmol·kg^−1^·min *†
de Lade et al., 2016 [3]	Strength Training: *N* = 5; Age = 57 ± 12 years; BMI = 27 ± 3 kg/m^2^; Aerobic Training: *N* = 6; Age = 54 ± 9 years; BMI = 36 ± 10 kg/m^2^.	Combined aerobic and strength	20 weeks, 3 times/week	Aerobic Training: 60–70% of maximum heart rate; Strength Training: moderate intensity, based on Borg scale 11–13	1. HbA1c: Aerobic group: Decreased from 8.6 ± 2.5 to 7.5 ± 1.7% ** Strength group: Decreased from 9.2 ± 1.9 to 7.4 ± 0.9% ** 2. Average glycemic level: Aerobic group: Decreased from 202 to 171 mg/dL ** Strength group: Decreased from 217 to 165 mg/dL **
Enteshary, M., Esfarjani, F., & Reisi, J., 2019 [14]	26 female patients with type 2 diabetes, aged 35–45 years, were divided into high-intensity, moderate-intensity and control groups.	Combined aerobic and strength	Aerobic: 5 times/week for 8 weeks Strength: 2 times/week for 8 weeks	High-intensity aerobic: 70–89% HRmax for 75 min; Moderate-intensity aerobic: 55–69% HRmax for 30 min. Strength: 15 to 20 repetitions, 25% elasticity	1. Betatrophin: Significantly higher in high-intensity (1006 ± 565.99 µg/mL *†) compared to the moderate-intensity group and (517.34 ± 27.63 µg/mL *) control (400.7 ± 36.89 µg/mL *) after 8 weeks. 2. Insulin: Increased in all groups post-training *; but is lower in both high (14.45 ± 7.68 µg/mL †) and moderate (19.04 ± 8.67 µg/mL †) intensity groups compared to control group (27.57 ± 18.78 µg/mL) after 8 weeks.
Johansen et al., 2020 [7]	98 type 2 diabetes duration <10 years (aged: 54.8 ± 8.9 years; BMI: 25–40 kg/m^2^), divided into lifestyle intervention + standard care group and standard care group	Combined aerobic and strength	Lifestyle intervention (Combined aerobic and strength training): Aerobic exercise: 12 months. 5–6 times/week; Strength exercise: 2–3 times per week, with gradually increasing weight and repetitions; Concurrent dietary intervention aiming for a BMI of 25 kg/m^2^; Standard care: consisted of individual guidance on disease management, lifestyle advice and blinded regulation of medication following a pre-specified algorithm.	NA	Insulin sensitivity (Matsuda Index): Standard care group: Decreased from 2.03 (95% CI: 1.40–3.54) to 1.17 (95% CI: 0.99, 1.38) * Lifestyle intervention group: Decreased from 2.12 (95% CI: 1.57–2.83) to 1.43 (95% CI: 1.29, 1.59) *† 1. Insulin secretion (Insulinogenic index): Standard care group: Increased from 0.20 (95% CI: 0.12–0.35) to 1.21 (95% CI: 0.98, 1.50) * Lifestyle intervention group: Increased from 0.31 (95% CI: 0.18–0.42) to 1.32 (95% CI: 1.15, 1.51) * 2. β-cell disposition index (DI) Standard care group: Increased from 0.42 (95% CI: 0.26–0.75) to 1.35 (95% CI: 1.03, 1.78) * Lifestyle intervention group: Increased from 0.56 (95% CI: 0.36–0.89) to 1.90 (95% CI: 1.59, 2.27) *†
Mir et al., 2020 [33]	Combined exercise: *N* = 10; Age = 58.9 ± 3.54 years; BMI = 28.77 ± 3.12 kg/m^2^; Control: *N* = 9; Age = 57.7 ± 4.57 years; BMI = 28.21 ± 2.42 kg/m^2^.	Combined HIIT and strength	12 weeks; 3 times/week	HIIT: 3 × 4 min treadmill intervals at 70–90% HRmax, each followed by 3 min active recovery at 50–70% HRmax. Strength Training: 6 exercises at 55–80% 1RM: leg press, chest press, front thigh, lat pull-down, back thigh, shoulder press (upper and lower body).	1. HbA1c: Combined exercise group: decreased from 7.49 ± 0.86 to 6.97 ± 0.89% *† Control group: no change 2. Insulin: Combined exercise group: decreased from 7.67 ± 1.71 to 6.64 ± 1.47 IU/mL *† Control group: no change 3. HOMA-IR: Combined exercise group: decreased from 3.12 ± 1.06 to 2.33 ± 0.69 *† Control group: no change
Mancilla et al., 2021 [34]	32 participants with type 2 diabetes risk or diagnosed (aged: 58 ± 7 years; BMI > 26 kg/m^2^), divided into morning exercise group (*N* = 12) and afternoon exercise group (*N* = 20)	Combined aerobic and strength	3 times/week for 12 weeks	Aerobic: 2 times/week, 30 min at 70% Wmax (cycling); Strength: 1 time/week, 3 × 10 reps at 60% maximum voluntary contraction;	1. Rate of glucose disappearance: Morning group: Increased from −10 to +10 μmol/kgFFM/min Afternoon group: Increased from −5 to +15 μmol/kgFFM/min
Amaravadi et al., 2024 [35]	Exercise group: *N* = 75; Age 56.05 ± 8.77; Control group: *N* = 71; Age 53.90 ± 10.20.	Combined aerobic and strength	12 weeks; 3 to 5 times/week; 30–45 min/time	Aerobic: Warm-up: 10 min of light-intensity exercises including breathing exercises, upper limb exercises, heel raises, and stretching of Achilles tendon, hamstrings, and quadriceps; Walking Training: Treadmill walking; duration and intensity progressed based on a 6-Minute Walk Distance and Rating of Perceived Exertion; Strength Exercises: Target major muscle groups of the trunk, shoulder girdle, pelvic girdle, and upper and lower limbs; starting at 5 repetitions, 1–2 sets, progressively increased every 2 weeks.	1. HOMA-IR Exercise group: decreased from 4.81 ± 2.69 to 3.35 ± 1.82 * Control group: increased from 4.63 ± 2.69 to 5.38 ± 2.82 *† 2. Fasting insulin Exercise group: decreased from 12.67 ± 5.92 to 9.49 ± 4.64 μU/mL * Control group: increased from 12.15 ± 6.15 to 13.14 ± 6.39 μU/mL *† 3. Fasting blood sugar: Exercise group: decreased from 159.55 ± 33.59 to 136.81 ± 18.11 mg/dL *; Control group: no change † 4. Postprandial blood sugar: Exercise group: decreased from 210.2 ± 58.29 to 183.8 ± 44.1 mg/dL * Control group: no change † 5. HbA1c: Exercise group: decrease from 8.11 ± 1.27 to 7.52 ± 1.05% Control group: increase from 8.06 ± 0.73 to 8.45 ± 0.80% †

* Indicate within-group differences (* = *p* < 0.05, ** = *p* < 0.01); † indicates between-group differences (*p* < 0.05). HbA1c: Glycated hemoglobin; AUC: area under the curve; DI: Disposition Index; VO_2_peak: Peak Oxygen Uptake; HOMA-IR: Homeostatic Model Assessment of Insulin Resistance; Rd glucose: Rate of disappearance of glucose; NS: Not significant.

**Table 4:** Studies reviewed on Strength Exercise.

Reference	Subjects’ Characteristics	Exercise Intervention			Main Findings
		Type	Duration, Frequency	Intensity	
Misra et al., 2008 [36]	*N* = 30; Age = 40.8 ± 8.1 years; BMI = 24.1 ± 3.9 kg/m^2^	Strength	12-week, 3 times/week	Started < 3 RM, 2 sets × 10 reps; +0.5 kg/week if completed;	1. Fasting Blood Glucose: decreased from 10.07 ± 2.0 to 7.4 ± 1.2 mmol/L * 2. HbA1c (%): decreased from 7.7 ± 0.5 to 7.2 ± 0.3 mmol/L *.
Kwon et al., 2010 [37]	28 patients (Resistance training group *N* = 13, Control group *N* = 15); BMI = 27.4 ± 2.5 kg/m^2^; age = 56.4 ± 7.1 years.	Resistance	3 times/week for 12 weeks	Elastic band training at 40–50% 1RM; 3 sets per exercise (~60 min/session).	3. Insulin resistance: Resistance training group: no change; Control group: no change.

* Indicate within-group differences (* = *p* < 0.05, ** = *p* < 0.01). HbA1c: Glycated hemoglobin; NS: Not significant.

**Table 5:** Studies reviewed on HIIT Exercise.

Reference	Subjects’ Characteristics	Exercise Intervention			Main Findings
		Type	Duration, Frequency	Intensity	
Petersen et al., 2024 [6]	Type 2 diabetes group: *N* = 15, aged 40–65 years, BMI 27–36; Obese control group: *N* = 15, age-matched, BMI 27–36; Lean control group: *N* = 18, age-matched, BMI 20–25.	HIIT	8 weeks, 3 times/week	Each training session included training blocks of 5 times 1 min training at high intensity (≥85% of HRmax), interspersed with a 1 min period of active or passive recovery	1. Disposition index (DI) (AIRg × GDR, clamp/L): Type 2 diabetes group: Increased from 4.7 ± 2.5 to 15 ± 6 **† Obese control group: Increased from 171 ± 22 to 218 ± 30 * Lean control group: Increased from 181 ± 25 to 237 ± 40 (clamp/L) * 2. Insulin, basal: Type 2 diabetes group: Decreased from 106 ± 21 to 79 ± 12 pmol/L *† Obese control group: No change Lean control group: No change 3. Insulin GDR, clamp (mg/min/m^2^): Type 2 diabetes group: Increased from 210 ± 24 to 317 ± 36 **† Obese control group: Increased from 351 ± 26 to 447 ± 29 * 4. Lean control group: Increased from 356 ± 30 to 463 ± 36 *

* Indicate within-group differences (* = *p* < 0.05, ** = *p* < 0.01); † indicates between-group differences (*p* < 0.05). HIIT: High-intensity interval training; GDR: Glucose Disposal Rate; DI: Disposition Index; NS: Not significant.

## Data Availability

No new data were created or analyzed in this study.

## Supplementary Materials

The following supporting information can be downloaded at: https://www.mdpi.com/article/10.3390/metabo15110739/s1, Table S1: Search strategy for web of science.; Table S2: Search strategy for PubMed.
