# Supplementary material for: Optimizing Exercise for Type 2 Diabetes Management: Comparative Insights from Aerobic, Resistance, Interval and Combined Training Protocols
Source: Metabolites. 2025 Nov 12;15(11):739. doi: 10.3390/metabo15110739 (PMC12654782; doi:10.3390/metabo15110739)
Supplement: Supplementary file 1 [file metabolites-15-00739-s001.zip › metabolites-3945060-supplementary.pdf]

Table S1. Search strategy for web of science.

Filter:	(English) AND (in Title)	Results
#1	(exercise*) AND (insulin secretion) AND (type 2 diabetes)	383
#2	(exercise*) AND (insulin secretion" OR "insulin sensitivity" OR "β-cell function" OR HOMA-IR OR HbA1c OR "glycemic control" OR "glucose control") AND (type 2 diabetes)	169
#3	Total after merging duplicate articles from #1 and #2	456

Table S2. Search strategy for PubMed.

Filter:	(English) AND Title/Abstract)	Results
#1	(exercise*) AND (insulin secretion) AND (type 2 diabetes)	219
#2	(exercise*) AND (insulin secretion" OR "insulin sensitivity" OR "β-cell function" OR HOMA-IR OR HbA1c OR "glycemic control" OR "glucose control") AND (type 2 diabetes)	4
#3	Total after merging duplicate articles from #1 and #2	217
